# Biological Effects of Green Tea Capsule Supplementation in Pre-Surgery Postmenopausal Breast Cancer Patients

**DOI:** 10.3389/fonc.2013.00298

**Published:** 2013-12-13

**Authors:** Steven S. Yu, Darcy V. Spicer, Debra Hawes, Chiu-Chen Tseng, Chung S. Yang, Malcolm C. Pike, Anna H. Wu

**Affiliations:** ^1^Department of Medicine, Keck School of Medicine, University of Southern California, Los Angeles, CA, USA; ^2^Department of Pathology, Keck School of Medicine, University of Southern California, Los Angeles, CA, USA; ^3^Department of Preventive Medicine, Keck School of Medicine, University of Southern California, Los Angeles, CA, USA; ^4^Department of Chemical Biology, Ernest Mario School of Pharmacy, Rutgers University, Piscataway, NJ, USA; ^5^Department of Epidemiology and Biostatistics, Memorial Sloan-Kettering Cancer Center, New York, NY, USA

**Keywords:** chemoprevention, green tea, postmenopausal breast cancer

## Abstract

Regular green tea intake has been associated with an inverse risk of breast cancer. There are compelling experimental evidence that green tea, particularly, epigallocatechin gallate, the most potent green tea catechin, possesses a range of anti-cancer properties. We conducted a pre-surgical study of green tea capsules vs. no-green tea in women with primary breast cancer to determine the effects of green tea supplementation on markers of biological response. Postmenopausal women with ductal carcinoma *in situ* (DCIS) or stage I or II breast cancer took green tea capsules (940 mg per day) for an average of 35 days prior to surgery (*n* = 13) or received no green tea (*n* = 18). Paired diagnostic core biopsy and surgical specimen samples were analyzed for cell proliferation (Ki-67), apoptosis (caspase-3), and angiogenesis (CD34) separately in benign and malignant cell components. There were no significant changes in caspase-3 and CD34 in the green tea and no green tea groups and there were no significant differences in the change in these markers between the two groups. However, Ki-67 levels declined in both benign and malignant cell components in the green tea group; the decline in Ki-67 positivity in malignant cells was not statistically significant (*P* = 0.10) but was statistically significant in benign cells (*P* = 0.007). Ki-67 levels in benign and malignant cells did not change significantly in the no green tea group. There was a statistically significant difference in the change in Ki-67 in benign cells (*P* = 0.033) between the green tea and the no green tea groups. The trend of a consistent reduction in Ki-67 in both benign and malignant cells in the green tea group warrants further investigations in a larger study of breast cancer patients or high-risk women.

## Introduction

Worldwide, breast cancer is the most common invasive cancer in women, accounting for nearly one fourth of all cancers in women. In 2010, nearly 1.6 million women worldwide were diagnosed with breast cancer, accounting for ∼14% of the cancer deaths in women. The incidence of breast cancer is highest in North America and Europe and lowest in Asia (1, 2). The historically lower incidence in Asian populations have been attributable, in part, to later ages of menarche, low body weight particularly in postmenopausal women, infrequent use of menopausal hormones, as well as to lifestyles factors including physical activity, and regular intake of soy based foods, plant-rich diet, and green tea (3). In a meta-analysis we conducted that included 5,604 breast cancer cases and 5,487 control women, a significant inverse association between green tea consumption and breast cancer incidence was found although a similar risk reduction was not found in prospective cohort studies which included non-daily or non-weekly tea drinkers in the baseline group. This difference in the definition of unexposed group between the prospective and case-control studies may have contributed, in part, to the differences in results (4). In two Japanese cohort studies, high daily green tea intake among patients with breast cancer has been associated with a decrease in risk of recurrence and mortality (5, 6).

Tea is the most widely consumed beverage in the world. Green tea, made from the leaves of the Camellia sinensis plant, accounts for ∼20% of the world’s tea production; and is the main tea consumed in Japan and China. Green tea extract is rich in antioxidant polyphenols most notably epigallocatechin-3-gallate (EGCG). EGCG has an antioxidant activity about 25 and 100 times greater than that of vitamins E and C, respectively, and is the most potent of all the catechins (7). Though the effects are relatively small, green tea polyphenols have been found to favorably influence several markers of breast cancer risk such as circulating estrogens, androgens, and mammographic density (4). Strong experimental evidence shows that EGCG influences cell growth and inhibits cell proliferation and angiogenesis, and induces apoptosis of preneoplastic and neoplastic cells, inhibiting essential mechanisms of cancer cell survival in different organ sites of various animal models and cell lines (8). In an effort to understand green tea’s effects on breast tissue, we conducted standard immunohistochemical (IHC) analysis of markers of cell proliferation (Ki-67), apoptosis (cleaved caspase-3 (casp-3), and angiogenesis (CD34) in patients diagnosed with breast cancer to investigate the short-term clinical effects of green tea supplementation.

## Materials and Methods

### Study design

The study was a pre-surgical trial of a green tea capsule vs. no capsule in postmenopausal women conducted at the Los Angeles County-University Southern California Medical Center (LAC-USC) between 2008 and 2009. The study included patients with ductal carcinoma *in situ* (DCIS) or primary invasive stage I or II breast cancer (as classified after diagnostic biopsy and subsequently confirmed at definitive surgery). There were 13 patients in the green tea group (1 DCIS and 12 invasive cancers). As a comparison group, we included six patients who declined to participate in the green tea intervention and nine other patients who had the necessary tissue specimens available for study (5 DCIS and 10 invasive cancers).

### Study capsules

Breast cancer patients in the green tea group took three green tea capsules daily (Pro Health Green Tea Mega EGCG^®^, 725 mg/capsule). The control group did not receive any capsules. Prior to the initiation of this pilot study, the capsules were tested in the laboratory of Dr. Chung S Yang (see Materials and Methods below) and were found to contain very comparable amounts of tea catechins; each 725 mg capsule contained ∼314 mg EGCG. Thus women in the green tea group consumed ∼940 mg EGCG per day (equivalent to ∼8–10 cups of green tea) between their breast cancer diagnoses and their surgery date. The average duration of green tea intake was 35 days for women in the green tea group. The dates of surgery were decided independent of and uninfluenced by participation in the study.

### Study procedures

Postmenopausal women at the LAC-USC with a suspicious mammogram necessitating a diagnostic core biopsy or those with biopsy proven breast cancer awaiting definitive treatment were approached to participate in this study. Briefly, postmenopausal women who were non-tea drinkers of any kind (i.e., they consumed less than a cup per week of green tea, black tea, or herbal tea) were asked if they were willing to take green tea capsules for ∼3 weeks before surgery or to serve in the control group which received no green tea supplementation. Willing participants were asked to consent to the use of their diagnostic core biopsy and surgical specimens for studies of cell proliferation and other markers (see Immunohistochemical Analysis). Blood specimens from subjects willing to take green tea capsules were drawn and tested to verify eligibility status which required having normal liver function (aspartate aminotransferase, alanine aminotransferase, and alkaline phosphatase). Baseline urine specimens were collected before initiation of use of green tea capsules and on the day of definitive surgery and were tested for tea catechin levels (see below). Participants were contacted by telephone at least every 7 days; those in the green tea group were asked if there were any side effects associated with taking the tea capsules and those in the control group were asked to confirm that they were not drinking any tea.

### Urinary tea catechin measurement

Urine samples from participants were identified by unique codes and were assayed in a single batch. Urinary concentrations of various green tea catechins and metabolites including epigallocatechin (EGC), and 4′-*O*-methyl-epigallocatechin (MeEGC) were determined in the laboratory of Dr. Yang analyzed by liquid chromatography/electrospray ionization tandem mass spectrometry with data-dependent acquisition (9, 10). Urinary creatinine (Cr) level was determined on each sample using standard methods.

### Immunohistochemical analysis

**Figure 1:**
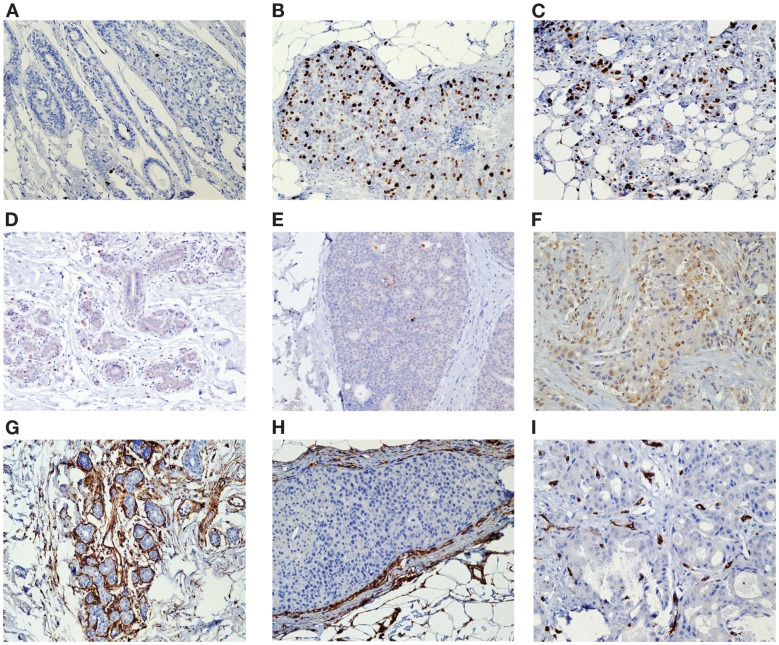
**Tissue was stained for specific markers of cell proliferation (Ki-67), apoptosis (casp-3), and angiogenesis (CD34) using the respective antibodies**. Separate IHC analyses were performed on the benign and malignant (DCIS and invasive) breast cell components Ki-67 Histology of biopsy benign **(A)**, DCIS **(B)**, and invasive tissue **(C)** Caspase-3 Histology of biopsy benign **(D)**, DCIS **(E)**, and invasive tissue **(F)** CD34 Histology of biopsy benign **(G)**, DCIS **(H)**, and invasive tissue **(I)**.

We conducted standard IHC analysis of the formalin-fixed paraffin-embedded (FFPE) samples of the core biopsies and surgically removed breast tissues (referred hereafter as surgical specimens). Multiple adjacent FFPE sections were cut at 5 μm, deparaffinized, and hydrated. All slides were subjected to antigen retrieval as described in our previous studies (11, 12) and were scored by the study pathologist (DH) in a blinded manner. The slides were stained for specific markers of cell proliferation (Ki-67), apoptosis (casp-3), and angiogenesis (CD34) using the respective antibodies: Ki-67, clone MIB-1, 1:200 dilution (Dako Inc., Carpinteria, CA, USA); cleaved caspase-3, clone (D175) (5A1E), 1:100 dilution (Cell Signaling Technology, Inc., Danver, MA, USA); and CD34, clone QBEnd, prediluted (Leica Microsystems, Buffalo Grove, IL, USA). Separate IHC analyses were performed on the benign and malignant (DCIS and invasive) breast cell components (Figure 1). Benign and malignant cells were separated visually based on morphological criteria and characteristics. The benign cells were adjacent to the tumor and were morphologically consistent with normal breast epithelium. DCIS cells were morphologically malignant but were contained within the basement membrane and did not show evidence of invasion whereas the invasive malignant cells were seen to invade into the breast stroma. Malignant cells have markedly increased numbers of large cells that expand the terminal ductal lobular units and extend to the ducts with enlarged hyperchromatic nuclei and decreased nuclear to cytoplasmic ratios. Necrosis was commonly but not always seen in malignant cells when compared with benign cells.

Automated methods were used to assess Ki-67 and CD34. The slides were assessed using the Automated Cellular Imaging System (ACIS II™) (Aliso Viejo, CA, USA) which we have used successfully in previous studies (11, 12). Using this system, epithelial cells were determined to be negative or positive based on color (DAB positive, hematoxylin negative) via the appropriate software applied to digitized images. For Ki-67, the nuclear area of positive epithelial cells vs. the nuclear area of all cells was used by the ACSI software to determine the percentage positive. For CD34 the ACIS microvessel density application was used to assess the CD34-positive fibroblast and endothelial cells divided by the tissue area. For caspase-3, which is a non-nuclear marker, we used standard light microscopy manual counting methods. Staining intensity was scored as 0–1 (weak staining), 2 (moderate staining), and 3 (strong staining). Cells with staining intensity score of 2 or greater were considered positive.

### Data analysis

Mean levels of the three biomarkers (Ki-67, casp-3, CD34) within cell type components (benign vs. malignant cells) and sample type (core biopsy vs. surgical specimens) were compared between treatments using the student two-sample *t*-tests as well as the ranksum test. The difference in the change in these biomarkers between core and surgical sample was also compared between treatments using both the *t*-test and ranksum test, for each cell type component. In addition, the change in biomarkers between core and surgical samples, within cell type and treatment, was compared using both a paired *t*-test and signed rank test. Both parametric (*t*-test) and non-parametric (ranksum, signrank) tests were performed and shown in the tables to gage the robustness of the observed results. Tests which were statistically significant on both tests were viewed as more likely representing a real effect. *P* values <0.05 were considered statistically significant and all *P* values quoted are 2-tailed. All analyses were conducted using the statistical software package STATA 11 (STATA Corporation, College Station, TX, USA).

## Results

Paired diagnostic core biopsy and surgical specimen samples were available on 28 breast cancer patients, 13 consumed green tea capsules, and 15 were in the control group (Table 1). The majority of patients in both groups were Latina: 92% Latinas in the green tea group and 60% in the control group (*P* = 0.07). Neither ER/PR status nor stage at diagnosis differed between the two groups. In tested patients before treatment, baseline urinary tea catechin levels were low and did not differ between the control and green tea groups (e.g., levels of MeEGC were <0.01 μmol/g Cr in both groups). Urinary tea catechin concentrations in the samples from the non-green tea patients remained low throughout the study but this was not measured in the historical controls, while the urinary tea catechin levels from the green tea group increased significantly by 2–10 fold for different catechins (e.g., MeEGC was 7.2 μmol/g Cr in green tea group) after an average of 35 days of supplementation.

**Table 1:** **Demographic and clinical characteristics of study participants with diagnostic core biopsy and surgical specimen samples**.

	Green tea (*n* = 13)	Control (*n* = 15)
Mean (SD) age	61.0 (4.9)	59.3 (6.8)
Race
Latina American	12	9
African American	0	5
Asian	1	1
Estrogen receptor (ER)
Positive	9	10
Negative	4	5
Progesterone receptor (PR)
Positive	6	7
Negative	7	8
ER/PR combined
ER^+^PR^+^	6	7
ER^+^PR^−^	3	3
ER^−^PR^−^	4	5
Stage
DCIS	1	5
I	6	4
II	6	6

There were significant differences in the levels of two of the three biomarkers between benign and malignant cells in the core biopsy samples. Ki-67 positivity in malignant cell components (22.8%, SE = 2.4%) was significantly higher than the levels in benign cell components (4.4%, SE = 2.5%) (*P* < 0.0001). Caspase-3 positivity in malignant cell components (13.7%, SE = 2.8%) was non-significantly higher than levels in benign cell components (8.4%, SE = 3.2%) (*P* = 0.22). Levels of CD34 immunoreactivity were significantly higher in the benign cell components (1.43%, SE = 0.22%) than in the malignant cell components (0.33%, SE = 0.19%) (*P* < 0.0001).

**Figure 2:**
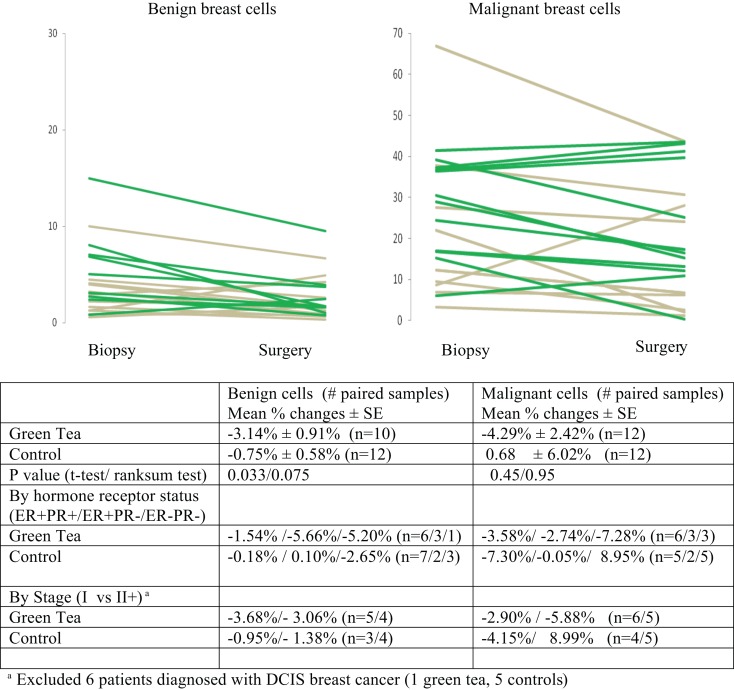
**Mean percent changes (±standard error of means) in quantitative Ki-67 immunohistochemical staining between diagnostic core biopsy and surgical specimens (green lines for green tea; gray lines for control); all subjects and by hormone receptor status and stage at diagnosis**.

**Figure 3:**
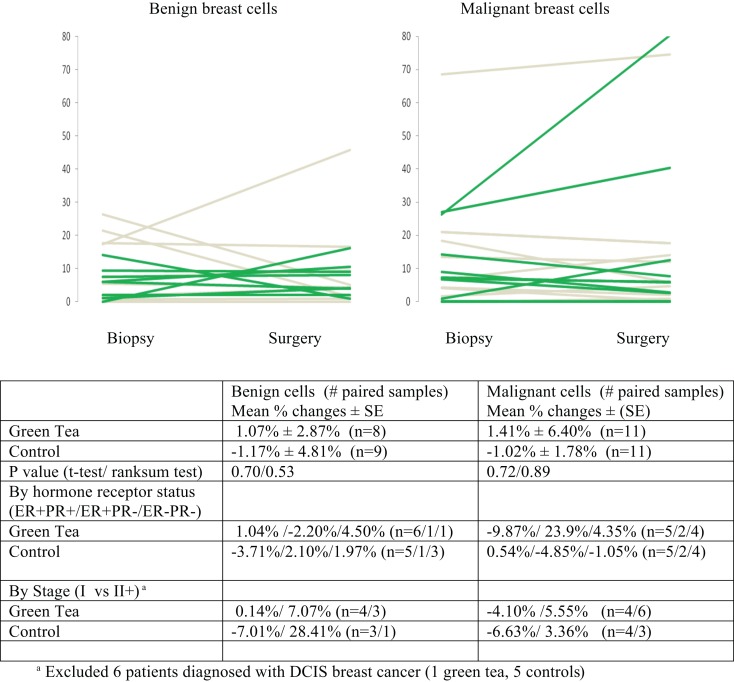
**Mean percent changes (±standard errors of mean) in quantitative caspase-3 immunohistochemical staining determined using diagnostic core biopsy and surgical specimens (green lines for green tea; gray lines for control); all subjects and by hormone receptor status and stage at diagnosis**.

**Figure 4:**
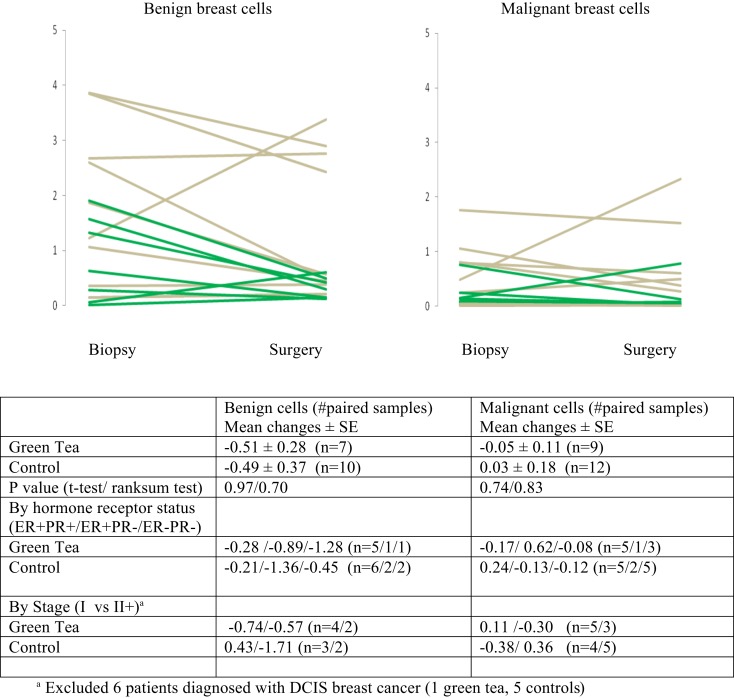
**Mean changes (±standard errors of mean) in quantitative CD34 immunohistochemical staining between diagnostic core biopsy and surgical specimen samples (green lines for green tea; gray lines for control); all subjects and by hormone receptor status and stage at diagnosis**.

IHC data are summarized in Figures 2–4, showing changes in Ki-67, caspase-3, and CD34 for individual patients according to treatment group separately for benign and malignant cell components (Figures 2–4). In the green tea group, reductions in Ki-67-positivity were observed consistently in benign cells (−3.14%, *P* = 0.007) and malignant cells (−4.29%, *P* = 0.10); the reduction in benign cell components was statistically significant (Table 2, top). In the control group, changes in Ki-67 positivity in benign cells (−0.75%, *P* = 0.22) and malignant cells (+0.68%, *P* = 0.91) were slight. The decrease in Ki-67 positivity in benign cells in the green tea group differed significantly from the change in the control group (*P* = 0.033) but the changes in malignant cell components did not differ significantly between the green tea and control groups (*P* = 0.45) (Figure 2). Results were similar when we used non-parametric tests. The decrease in Ki-67 positivity in benign cells and malignant cell components was found consistently by ER/PR positivity status (ER^+^PR^+^, ER^+^/PR^−^, ER^−^PR^−^) and tumor stage at diagnosis (Stage I and II+) in the green tea group but not in the control group although we did not perform formal statistical testing of differences by these subgroups because of small numbers (Figure 2).

**Table 2:** **Diagnostic core biopsy and surgical specimen mean percent levels (±standard error of mean) of Ki-67, caspase-3, and CD34 in benign and malignant cell components in green tea and control group**.

Ki-67		Green tea (*n* = 10)	Control (*n* = 12)	*P* value^c^	*P* value^d^
Benign cells	Biopsy	5.95 ± 1.30	3.05 ± 0.74	0.057	0.075
	Surgical	2.81 ± 0.81	2.30 ± 0.57	0.61	0.60
	Change	−3.14 ± 0.91	−0.75 ± 0.58	0.033	0.075
	*P* value^a^^,^^b^	0.007/0.11	0.22/0.21		

		**Green tea (*n* = 12)**	**Control (*n* = 12)**		

Malignant cells	Biopsy	27.47 ± 3.31	18.13 ± 5.39	0.15	0.04
	Surgical	23.18 ± 4.31	18.81 ± 6.41	0.58	0.30
	Change	−4.29 ± 2.42	0.68 ± 6.02	0.45	0.95
	*P* value^a^^,^^b^	0.10/0.14	0.91/0.18		

**Cleaved caspase-3**		**Green tea (*n* = 8)**	**Control (*n* = 9)**	***P* value^c^**	***P* value^d^**

Benign cells	Biopsy	5.72 ± 1.65	10.86 ± 3.31	0.20	0.47
	Surgical	6.79 ± 1.81	9.69 ± 4.82	0.60	0.92
	Change	1.07 ± 2.87	−1.17 ± 4.81	0.70	0.53
	*P* value^a^^,^^b^	0.72/0.51	0.81/1.00		

		**Green tea (*n* = 11)**	**Control (*n* = 11)**		

Malignant cells	Biopsy	13.16 ± 3.47	14.22 ± 6.01	0.88	0.45
	Surgical	14.57 ± 7.42	13.20 ± 6.42	0.89	0.90
	Change	1.41 ± 6.40	−1.02 ± 1.78	0.72	0.89
	*P* value^a^^,^^b^	0.83/0.63	0.58/0.63		

**CD34 (#vessels/tissue area)**		**Green tea (*n* = 7)**	**Control (*n* = 10)**	***P* value^c^**	***P* value^d^**

Benign cells	Biopsy	0.83 ± 0.29	1.85 ± 0.43	0.09	0.12
	Surgical	0.32 ± 0.07	1.36 ± 0.42	0.058	0.12
	Change	−0.51 ± 0.28	−0.49 ± 0.37	0.97	0.70
	*P* value^a^^,^^b^	0.12/0.13	0.22/0.24		

		**Green tea (*n* = 9)**	**Controls (*n* = 12)**		

Malignant cells	Biopsy	0.18 ± 0.08	0.45 ± 0.16	0.18	0.62
	Surgical	0.13 ± 0.08	0.48 ± 0.21	0.18	0.39
	Change	−0.05 ± 0.11	0.03 ± 0.18	0.74	0.83
	*P* value^a^^,^^b^	0.66/0.11	0.88/0.24		

*^a^*P* value for changes between biopsy core and surgical specimen samples for benign or malignant cells separately for green tea and control groups using paired *t*-test*.

*^b^*P* value for changes between biopsy core and surgical specimen samples for benign or malignant cells separately for green tea and control groups using signed rank test*.

*^c^*P* value for differences between levels in green tea and control groups using *t*-test*.

*^d^*P* value for differences between levels in green tea and control groups using ranksum test*.

There were no significant changes in caspase-3 levels in benign and malignant cell components between core biopsy and surgical specimen samples in the green tea and control groups (Figure 3, Table 2-middle). Similarly, there were no significant changes in CD34 levels between core biopsy and surgical specimen samples in the green tea and non-green tea groups (Figure 4, Table 2-bottom). Subgroup analyses by ER/PR positivity status and tumor stage (I vs. II) at diagnosis did not reveal any consistent patterns in the changes in caspase-3 and CD34 levels in the green tea and control groups (Figures 3 and 4).

## Discussion

In an effort to understand the biological effects of green tea on breast cancer, we investigated the effects of green tea supplementation on markers of cell proliferation, apoptosis, and angiogenesis using paired pre-surgery (diagnostic core biopsy) and surgery specimens from 28 newly diagnosed breast cancer patients. Patients in the green tea group displayed a consistent pattern of reductions in markers of cell proliferation (Ki-67) in benign and malignant cell components; this was not observed in the no-green tea group. The Ki-67 reductions in the green tea group were found consistently irrespective of ER/PR status and stage of breast cancer at diagnosis. The change in Ki-67 in benign cells differed between the two groups; this was observed using both parametric and non-parametric tests although the change was borderline statistically significant using non-parametric test. No significant differences were found in changes in apoptosis (caspase-3) or angiogenesis (CD34) between the two groups.

Our finding that green tea supplementation can potentially decrease cell proliferation in human breast tissue, a fundamental hallmark of cancer cells, is noteworthy (13). Ki-67, a marker for proliferation, has been extensively studied as a prognostic marker in breast cancer and predictor of response to chemotherapy, and used in recurrence indexes (14). High Ki-67 in residual disease following neoadjuvant therapy has been associated with poorer long-term outcomes including overall survival in systematic reviews (15, 16). Use of chemotherapy, endocrine, and signaling agents have also been shown to lead to reductions in Ki-67 further supporting its ability to predict treatment benefit and to serve as a marker for long-term outcomes in operable breast cancer (17). Such evidence led to the recommendation of the International Ki-67 in Breast Cancer Working Group to conclude that measures of proliferation could be important both in standard clinical practice and, particularly, within clinical trials (14).

This study represents one of three controlled green tea intervention studies that have been conducted in breast cancer patients. Our study was a pre-surgical study whereas the previous two studies were conducted in breast cancer patients at least 6–12 months after they have completed standard treatment including chemotherapy and radiation (18, 19). Crew et al. conducted a phase IB randomized, double-blinded, placebo-controlled, dose escalation trial of a 6-month intervention of Polyphenon E (enriched green tea extract) in which breast cancer patients with hormone receptor negative tumors received 800, 1,200, or 1,600 mg EGCG daily (*n* = 26) or matching placebo capsules (*n* = 8) for 6 months. Breast tissue cell proliferation (Ki-67) was measured in random core biopsy obtained at baseline and after the 6-month intervention. At a similar or high dosage than our study, Ki-67 increased non-significantly after 6 months of intervention in both green tea and placebo groups although the change in proliferation did not differ significantly between the green tea and placebo groups (19). Our study included only postmenopausal women whereas the Crew et al. study included both pre-menopausal and postmenopausal breast cancer patients. Although Crew et al. obtained breast biopsies collected during days 7–14 of the menstrual cycle in the about 25% pre-menopausal women, it is difficult to be certain that these measurements were timed exactly at the same point in the menstrual cycle at baseline and at the end of 6 months of intervention. In another study, patients with invasive breast cancer (stage I–III) were randomized to drink decaffeinated green tea (*n* = 23) or herbal tea (*n* = 19) four times per day (∼960 ml/day) for 6 months. This study investigated changes in weight, body composition, and blood lipid and glucose-related biomarkers but did not assess biomarkers in breast tissues as study endpoints (18).

Other studies have been conducted to investigate the role of green tea supplementation on markers of biological response in cancers. Results from a pre-surgical study of green tea and prostate cancer are of particular interest. Nguyen et al. conducted a randomized, double-blind, placebo-controlled trial of Polyphenon E in men with prostate cancer who were scheduled to undergo radical prostatectomy. Similar to our study, this trial was designed to determine the effects of short-term supplementation (3–6 weeks before surgery) of green tea capsules (800 mg EGCG) (*n* = 24) compared to placebo capsules (*n* = 22) on changes in Ki-67, caspase-3, and CD34 levels. Although there were no significant changes in these three biomarkers between the two groups, there was a trend of reduction in Gleason score, and larger reductions in PSA levels and oxidative DNA damage in the green tea group (20).

Our findings of reduction in cell proliferation Ki-67 in association with green tea supplementation are consistent with a large body of experimental evidence that EGCG, the major component in green tea, inhibits tumor growth in numerous cancers including skin, gastrointestinal, prostate, cervical, breast, lung, and other cancers (21). In breast cancer cells, green tea or EGCG, have been found to inhibit cell proliferation, induce apoptosis, and block angiogenesis in cell cultures through multiple signaling pathways (22, 23). As we have summarized previously, numerous studies have investigated the effects of a variety of green tea products including green tea mixtures as well as specific catechins on mammary cancer using different rodent models. Oral administration of green tea using mouse xenograft models have demonstrated beneficial results including delaying mammary tumor onset, reducing the tumor burden, and reducing the number of invasive tumors. A study using the C3(1)/SV40 mouse model which more closely mimics the development and progression of human breast cancers found that administration of 0.5% Polyphenon E in drinking water inhibited mammary tumor growth. (24). Additional studies have suggested that the combination of green tea and tamoxifen is more potent than either agent alone in suppressing breast cancer growth in mice experiments (25), EGCG enhances tamoxifen-induced cellular apoptosis in ERα-negative MDA-MB-231 (26), and inhibits proliferation of trastuzumab-resistant human breast cancer cells (27). More recently, EGCG was found to reactivate ERα expression in ERα-negative MDA-MB-213 breast cancer cells via epigenetic mechanisms. This effect appeared to be enhanced when EGCG was combined with a histone deacetylase inhibitor (28). Promising results from these experimental studies emphasized the need to better understand the anti-cancer mechanisms associated with green tea in human studies.

Several limitations of the current study should be noted. In particular, this study was not randomized or blinded and had a very modest sample size. In addition, the duration of green tea intervention was relatively short (average of 35 days) and participants in the control group did not take a comparable placebo capsule. The biopsy Ki-67 levels were somewhat higher in the green tea than control group suggesting the possibility of imbalance between the two groups. Notwithstanding, the present study suggests that green tea supplementation may decrease proliferation of breast tissue although we found little evidence for its effect on apoptosis and angiogenesis. With the growing acceptance of Ki-67 as a prognostic factor and biomarker, finding agents that decrease cell proliferation and may potentially improve recurrence and survival remains a priority. The results of green tea supplementation are intriguing and warrant future investigation and validation by larger, randomized studies with longer term follow-up, in order to draw a more solid conclusion whether green tea products benefit breast cancer patients or high-risk women, as well as whether Ki-67 decline does indicate improved outcomes (e.g., reduction of disease recurrence, overall survival). Further studies on such biomarkers will provide valuable data to the development of novel treatments in breast cancer.

## Conflict of Interest Statement

The authors declare that the research was conducted in the absence of any commercial or financial relationships that could be construed as a potential conflict of interest.
